# Relationship between timeliness of contact and length of stay in older and younger patients of a consultation-liaison psychiatry service

**DOI:** 10.1192/pb.bp.114.047340

**Published:** 2015-06

**Authors:** Rebecca Wood, Anne P. F. Wand, Glenn E. Hunt

**Affiliations:** 1 Sydney Local Health District; 2 Sydney Medical School, University of Sydney; 3 South Eastern Sydney Local Health District; 4 University of New South Wales, Australia

## Abstract

**Aim and methods** The aims were to determine whether the timeliness of contact with a consultation-liaison psychiatry (CLP) service is associated with shorter lengths of stay (LOS), whether this relationship persists for stays greater than 4 days and whether this association varies with age. The length of stay was correlated with the time from admission to contact with the service (the referral lag (REFLAG)), and the REFLAG’s proportion of length of stay (REFLAG/LOS) for all 140 in-patients, those with stays greater than 4 days, and for those under and over 65 years.

**Results** The length of stay was significantly correlated with referral lag and logREFLAG/logLOS for all patients and for patients with stays greater than 4 days. The correlations remained significant for both age groups, but were stronger in the younger group.

**Clinical implications** Timeliness of contact with CLP was associated with shorter length of stay, particularly in younger patients. Psychiatric factors influencing length of stay in older patients should be studied by CLP services.

Consultation-liaison psychiatry (CLP) has not consistently demonstrated evidence of cost-effectiveness, which is partly related to the difficulty of conducting studies with robust methodology that reflect the real-life operation of CLP services.^1^ Some recent studies have demonstrated the effectiveness of intensive psychiatric consultation services with the specific brief of reducing length of stay (LOS).^2,3^ In contrast, studies of services with a traditional model of accepting referrals from a treating team, have found that earlier contact with CLP is associated with reduced lengths of stay.^4–11^ Most of these studies have compared length of stay with the proportion of the referral lag (i.e. REFLAG: the time from admission to patient contact with the CLP service) of the length of stay (i.e. REFLAG/LOS).^4–7,10^ This avoids directly comparing lengths of stay with the referral lag, as these are related variables. In support of this, REFLAG/LOS has been found to be independent of length of stay, if the stay is longer than 4 days.^10^ The primary aim of this study was to examine if the timing of contact is associated with length of stay for all patients referred to a CLP service, particularly when those with a stay less than 4 days were excluded. Furthermore, CLP services see many frail and complex older patients who utilise proportionally greater resources.^12^ Older in-patients with, or who develop, mental conditions while in hospital have been found to have longer lengths of stay.^13^ A secondary aim of this study was to compare the relationship of timeliness of referral and length of stay for patients under and over 65 years old.

## Method

### Participants

The study was conducted at a 215-bed acute metropolitan general hospital in Sydney, Australia. The CLP service at this hospital consisted of a part-time staff specialist psychiatrist (0.6 full-time equivalent) and a full-time psychiatry trainee. The hospital also employs mental health liaison nurses (0.9 full-time equivalent) who directly manage some ward referrals instead of the CLP service. The CLP service was referred 1.03% of all hospital in-patients during 2012, which is in keeping with previous studies.^14^ All consecutive in-patients referred to the CLP service, excluding those referred from the obstetrics and gynaecology department, from 1 January to 31 December, 2012, were included in the study. This exclusion was made because of a difference in referral pathway, as these patients had usually been seen on an ongoing basis during their out-patient antenatal care.

### Data collection

Data were collected for patients referred to the CLP service from the routinely used service referral forms and the medical record. Data collected included demographic information, admission date, date of first contact with CLP and number of contacts, length of stay, referring team, referral reason, medical diagnosis, psychiatric diagnosis, Karnofsky score on contact with the CLP service and at discharge. The Karnofsky score was recorded by the CLP team at initial contact and on discharge. This score was devised to quantify ability to carry out normal activities and self-care, and is rated from 0 (dead) to 100 (normal, no complaints, no evidence of disease) and has been established as a valid and reliable score of global functioning.^15^

Length of stay was calculated as the whole number of days from admission to the day of discharge from the hospital and if these were the same day this was counted as a length of stay of 1 day. The psychiatric diagnosis was made using the DSM-IV-TR criteria,^16^ and multiple psychiatric diagnoses were recorded with identification of the primary diagnosis relevant to the episode of care. This study was approved by the Human Ethics Committee for Sydney Local Health District (RPAH zone).

### Statistical analysis

Distributions were described as mean, standard deviation and range. The referral lag was calculated as the whole number of days between admission and first contact by the CLP service and this parameter was used to calculate the proportion of referral lag over length of stay (REFLAG/LOS). Thus, a REFLAG/LOS of 0.5 indicated that a patient was referred halfway through their admission and a REFLAG/LOS of 0.25, at the first quartile of the admission. Logarithmic transformations (logREFLAG/logLOS) were required because the data were positively skewed and logging the values made the data more normally distributed, consistent with previous studies.^4–7^ These variables were compared with the lengths of stay for all in-patient referrals, and then specifically in groups according to age (‘younger’ – defined as under 65 and ‘older’ – 65 years or more). Group differences were determined using one-way ANOVAs and Spearman’s correlations were used to assess associations between variables if any of the variables were not normally distributed. All data analyses were performed using SPSS version 17 for Windows. All *P*-values were two-tailed and significant differences between groups were determined using *P*<0.05.

## Results

There were 174 in-patient referrals to the CLP service in 2012. Of these, 34 were in-patients of the obstetrics service who were excluded from the analysis. The demographic profile, referring team, Karnofsky score and number of contacts by the CLP service of the remaining 140 patients are presented in Table 1. Patients 65 years and older were more likely to be born overseas, require an interpreter and have more contacts (reviews during admission) than younger patients. In total, seven (5%) in-patients died; four of these were less than 65 years old.

The most common referral reasons for all referrals were depression (45, 32%) and self-poisoning (18, 13%), followed by confusion (16, 11%) and medication review/past psychiatric history (14, 10%). For the two most common referral reasons, there was the greatest discrepancy in the age groups. There was a greater proportion of patients 65 years and older referred for depression (37, 41%) compared with those under 65 years of age (8, 16%); and a greater proportion of those with self-poisoning in the younger group (13, 26%) than the older group (5, 6%).

The most common medical diagnostic categories for all referrals were respiratory (21, 15%), oncological (18, 13%), related to self-poisoning (16, 11%) and neurological (15, 11%) conditions. Between the age groups the largest differences in percentage terms were for self-poisoning (12, 24% for the younger group and 4, 4% for the older group) and urosepsis (0, 0% *v.* 13, 14%).

The psychiatric diagnoses for both age groups are also presented in Table 1. There was a significant association between psychiatric diagnoses and age groups (χ^2^ = 14.804 (d.f. = 4), *P*<0.005). When the four diagnostic groups were assessed individually, it was noted that patients 65 years and over were more likely to have a diagnosis of a mood disorder than younger patients (χ^2^ = 5.20 (d.f. = 1), *P* = 0.23), whereas younger patients were more likely to have other diagnoses (*n* = 14, 28%), such as anxiety or adjustment disorders (χ^2^ = 7.59 (d.f. = 1) *P* = 0.006) compared with older patients (*n* = 9, 10%).

The average length of stay for all patients was 19.6 days, whereas the average hospital in-patient stay over the same time period was 3.5 days. The length of stay, referral lag and related parameters are detailed in Table 2. The lengths of stay for older patients was significantly longer than those patients who were under 65 years old (*F*(1,138) = 6.17, *P* = 0.014). There was also a significant age group difference for referral lag (*F*(1,138) = 4.80, *P* = 0.030) and logREFLAG/logLOS (*F*(1,124) = 4.41, *P* = 0.038).

When contact with the CLP service occurred during a 1-day admission (LOS = 1) or on day 1 of a longer admission (REFLAG = 0), this introduced a mathematical error in calculating the REFLAG/LOS and the logarithmic transformation of REFLAG/LOS. As the logarithm of zero is undefined and the logarithm of one is zero, these cases resulted in an undefined value for logREFLAG/logLOS. Therefore, these patients (*n* = 14) were not included in some analyses. There were no significant differences between the older and younger groups of patients in terms of the number of these cases.

The correlations between length of stay and the parameters related to referral lag are shown in Table 3. There were significant correlations between length of stay and all the referral lag parameters for all patients, which included the correlation between length of stay and referral lag when the values were logarithmically transformed (logREFLAG/logLOS, *r* = 0.38, *P* = 0.001). These relationships were more strongly correlated in patients under 65 years old. A total of 38 patients had a lengths of stay of 1-4 days. The positive correlation of the timeliness of referral and length of stay was only maintained for the REFLAG/LOS with logarithmic transformation (*r* = 0.242, *P* = 0.02) and the referral lag itself (*r* = 0.547, *P* = 0.001) when the 38 patients with a shorter length of stay were removed (Table 4).

**Table 1:** Demographic variables by age group

	Age 64 or younger (*n* = 50)	Age 65 or older (*n* = 90)	Total (*n* = 140)	*P*
Age, mean (s.d.)	43.6 (15.8)	79.2 (7.8)		

Women, *n* (%)	27 (54)	57 (63)	84 (60)	NS

Born overseas	21 (42)	62 (69)	83 (59)	0.002

Interpreter used	4 (8)	37 (41)	41 (29)	0.001

Department referred from, *n* (%)				
General medical	23 (46)	36 (40)	59 (42)	
Aged care and rehabilitation	1	32 (36)	33 (24)	
Intensive care unit	16 (32)	4 (4)	20 (14)	
Palliative care	4 (8)	9 (10)	13 (9)	
Emergency department	4 (8)	4 (4)	8 (6)	
Surgery	1 (2)	5 (6)	6 (4)	
Paediatrics	1 (2)	0	1 (0.7)	

Karnofsky score on admission, mean (s.d.)	24.8 (5.0)	25.1 (5.4)	25.0 (5.3)	NS

Karnofsky score on discharge, mean (s.d.)	61.4 (28)	54.1 (23)	57 (25)	NS

Contacts, mean (s.d.) range	4.4 (3.6) 1–18	7.6 (6.3) 1–41	6.4 (5.7)	0.002

Contacts per day after first contact, mean (s.d.)	1.24 (0.89)	0.95 (0.93)	1.05 (0.92)	NS

Psychiatric diagnosis,^a^ *n* (%)				
Organic brain disorder	12 (24)	35 (39)	47 (33.6)	NS
Mood disorder	6 (12)	26 (29)	32 (22.9)	0.023
No psychiatric diagnosis	10 (20)	12 (13)	22 (16)	NS
Psychotic disorder	8 (16)	8 (9)	16 (11)	NS
Other diagnoses^b^	14 (28)	9 (10)	23 (16)	0.006

Multiple psychiatric diagnoses, *n* (%)	9 (18)	22 (24)	31 (22)	NS

NS, not significant.

a. Chi-square, χ^2^ = 14.804 (d.f. = 4), *P* < 0.005.

b. Other diagnoses included: anxiety disorders, adjustment disorder, borderline personality disorder, somatoform disorders, substance misuse disorders, eating disorders and bereavement.

## Discussion

It is acknowledged that the average length of stay of patients with psychological comorbidity is much longer than the overall average length of stay.^17^ This is consistent with the results of this study where CLP-referred patients had a greater mean length of stay compared with the length for all patients at the studied hospital. It is therefore important to investigate factors that may relate to this disparity in stay length, which could then become targets of interventions to reduce healthcare costs. In this study, there is a significant association between early contact with CLP services and shortened stays for all patients referred to the CLP service. The association was strongest for patients under 65 years of age. The relationship was maintained for those patients with stays greater than 4 days.

The results regarding timeliness of contact with CLP services are in keeping with most previous studies.^4–11^ Only one study, which focused on patients with organic brain disorder referred to a CLP service, did not find that earlier referral predicted a shorter length of stay.^18^ Only two previous studies have separated out those patients with stays greater than 4 days.^3,10^ It could be argued that in a hospital stay less than 4 days, the impact of a CLP service is likely to be minimal given the frequently delayed response to psychiatric interventions, both pharmacological and psychological, and the multiple other factors that are involved in a patient’s readiness for discharge. Furthermore, it is particularly important for CLP services to demonstrate reduction in lengths of admissions in more complex long-term patients; where there is greater potential cost saving through shorter hospital stays.

None of the studies that have previously examined the impact on the length of stay of the proportion of the referral lag of the length of stay have specified the number of cases that have not been calculated because of the mathematical errors in those cases with a stay of 1 day, or who are referred on the same day of admission.^4–7^ This is not as important for those cases with a 1-day stay as there is no possibility that a CLP service could reduce this further. However, the necessity to not include those cases that are referred as early as possible does potentially reduce any positive effect demonstrated by CLP services using this measure. Thus, the number of cases that result in mathematical error should be reported in future studies.

### Differences between the two groups

There was a significant but comparatively weaker correlation between length of stay and timing of referral in older patients compared with the younger group, which was a disappointing finding as this is a large and important target group for CLP services. This result is in contrast to the study of the rapid assessment, interface and discharge integrated model (RAID), which found that most of the service’s cost savings were achieved through reduced lengths of stay and fewer readmissions in the geriatric wards.^2^ The authors suggested these outcomes were related to educating general hospital staff about mental health problems and efforts to link patients to appropriate pathways for community care.^2^ The difference in strength of correlation of the association found in the study presented here may be because of the inherent differences

**Table 2:** Comparison of length of stay (LOS) and referral lag (REFLAG) related parameters by age group

		Mean (s.d.) range			
	*n*	Age 64 or younger (*n* = 50)	Age 65 or older (*n* = 90)	Total (*n* = 140)	*P*
Length of stay, days		10.4 (10.2) 1–42	24.6 (39.5) 1–337	19.6 (32.9)	0.014

Referral lag	140	3.9 (5.0)	8.5 (14.5)	6.9 (12.2)	0.03

REFLAG/LOS	133^a^	0.498 (0.288)	0.408 (0.274)	0.441 (0.281)	NS

logREFLAG/log LOS	126^b^	0.405	0.533	0.490	0.038

NS, not significant.

a. Data for seven patients could not be calculated because of consultation on day of admission (REFLAG = 0).

b. Data for 14 patients could not be calculated because of a REFLAG = 0 (*n* = 7) or LOS = 1 (*n* = 7).

**Table 3:** Spearman’s correlations between referral lag related parameters and length of stay (LOS) by age group

	Length of stay, Spearman’s rho								
	Patients, 64 years and under			Patients 65, years and over			All patients		
Variable	*r*	*P*	*n*	*r*	*P*	*n*	*r*	*P*	*n*
Referral lag (REFLAG)	0.694	0.001	50	0.644	0.001	90	0.697	0.001	140

REFLAG/LOS	–0.530	0.001	48	–0.277	0.010	85	–0.378	0.001	133^a^

Log(REFLAG)/log(LOS)	0.565	0.001	42	0.228	0.037	84	0.380	0.001	126^b^

a. Data for seven patients could not be calculated because of consultation on day of admission (REFLAG = 0).

b. Data for 14 patients could not be calculated because of a REFLAG = 0 (*n* = 7) or LOS = 1 (*n* = 7).

between the two age groups, including the need for an interpreter. The study hospital serves an ethnically diverse population with 48.1% of the hospital’s catchment population born overseas, which explains the high level of utilisation of interpreters by this CLP service.^19^

The higher number of contacts with the CLP service received by the older group is likely reflective of the longer length of admission. This correlation has been found previously.^10^ It is unsurprising that patients who are in hospital longer will see CLP services on a greater number of occasions. In support of this, there was no significant difference between the two age groups in the average number of contacts/day after initial contact with CLP services, despite the greater need for interpreters in the older age group.

Surprisingly, the Karnofsky scores were not significantly different between the older and younger groups of patients, which would suggest that disparity in functional status does not account for the difference in the correlation results. This may reflect the limitations of this scale as it is most applicable to non-hospital-based supportive care settings, such as palliative care, rather than acute in-patient treatment.^20^

Previous studies have found factors that predict later referral to CLP services, such as higher social vulnerability,^12^ referral for depression and psychiatric diagnoses of adjustment disorder and delirium and no psychiatric diagnosis.^5^ Therefore, the profile of psychiatric diagnosis between the younger and older patients may also have contributed to the difference in impact of CLP on length of stay between the two groups. The older patients were more likely to be diagnosed with a mood disorder but,

**Table 4:** Spearman’s correlations between referral lag (REFLAG) related parameters and length of stay (LOS) when patients with a length of stay <4 days (*n* = 38) were excluded

Variable	*n*	Length of stay, *r*	*P*
Age	102	0.090	NS

Referral lag (REFLAG)	102	0.547	0.001

REFLAG/LOS	97^a^	–0.087	NS

Log(REFLAG)/log(LOS)	97^a^	0.242	0.02

NS, not significant.

a. Data for five patients could not be calculated because of consultation on day of admission (REFLAG = 0).

unexpectedly, there was no difference for the diagnoses related to organic brain disorders between the two age groups. There are two possible explanations for the relatively low frequency of referred patients diagnosed with cognitive disorders. First, the study hospital has been found to have low rates of recognition of cognitive disorders by referring teams.^21^ Second, a concurrent delirium-prevention study took place at this hospital that improved staff knowledge and confidence and reduced the occurrence of delirium, which may have reduced the overall number of individuals with cognitive disorders referred.^22^

There was a greater proportion of patients referred for, and diagnosed with, self-poisoning in the younger group of patients compared with the older group, which may have influenced the difference seen in the two age groups. Psychiatric input is almost universal in patients who are admitted with self-harm as the reason for the consultation is immediately obvious and this may be reflected in earlier referral of these patients by the treating team^5^ and arguably therefore, greater influence of the CLP team on management and discharge planning.

There were other significant differences in the older group of patients in this sample in terms of length of stay and referral lag. The fact that there was a longer length of stay in older patients referred to CLP is not surprising and is in keeping with previous reports.^13^ Three in-patients, all older than 65 years of age, had stays greater than 100 days. These outliers were included in the final statistical analysis but did not significantly influence results when removed. The longer admissions may have been because of factors such as waiting for residential care placement or rehabilitation, and greater medical comorbidity, which are less common in younger patients. Arguably, there is limited scope for CLP to influence lengths of stay when these factors are active.

The longer referral lag for the older patients is somewhat surprising for this service, which has a liaison attachment with the aged care and rehabilitation department. This department referred only 24% of all older (≥65) CLP patients; a greater proportion of older patients were referred instead by general medical teams (40%), which carry a larger total patient load. However, the longer referral lag in the older patients may also reflect other differences between the younger and older patient groups that influence timing of the referral from the teams. For example, proportionally more older patients were born overseas and required an interpreter. It is possible that delays in accessing interpreters precluded early referral to CLP.

### Limitations

This study was conducted in a district hospital with a representative sample of CLP patients. It did not involve alterations to the established CLP service or its referral patterns and was conducted retrospectively, which eliminated the possibility of the Hawthorne effect. Therefore, although based on small numbers, the results are generalisable to most CLP services, which are not designed as specialised acute intervention teams focused upon reducing lengths of stay.

The data collected included many of the parameters that may have contributed to the differences in effect of CLP contact on length of stay of the two groups. However, the re-admission rates of the two groups were not known and this has been suggested as an important potential consequence of reducing length of stay, although this is contested by some studies.^23^ No other study that has examined the timeliness of CLP contact has included this parameter,^4–7,10^ but it has been included in other cost-effectiveness studies with different methodology.^2^ This would be an important point for inclusion in future studies of timeliness of CLP contact and lengths of stay.

The limits of interpretation previously discussed regarding the association of timeliness of referral with lengths of stay also apply to this study. The demonstrated relationship between the time to referral and stay length cannot be assumed to be causal and it remains possible that the association is a result of unmeasured factors. These factors may include those associated with the request for consultation or also that the direction of the inference may be reversed.^24^ Thus, it is not possible to state that this CLP service directly shortens lengths of stay if there is greater proportional involvement in a patient’s admission, except to state that a positive association between these two variables has been demonstrated.

## Implications

Timeliness of referral was associated with shorter lengths of stay, including for those with stays of more than 4 days. This correlation was weaker for older than for younger patients. There are multiple and complex factors that likely lead to this result, particularly the greater likelihood of the older patients requiring an interpreter and being born overseas, as well as a greater delay in contact with CLP services and a longer length of stay when compared with younger patients. Given the ageing population, further exploration of these factors should be a priority for CLP services, as this is a group where CLP could have a considerable impact and cost-benefit. It is important to evaluate whether better outcomes achieved through hospital-wide education about mental health problems and emphasising clear pathways for community care can be replicated.^2^

## References

[1] Wood R Wand APF The effectiveness of consultation-liaison psychiatry in the general hospital setting: a systematic review. J Psychosom Res 2014; 76: 175–92. 24529036 10.1016/j.jpsychores.2014.01.002

[2] Tadros G Salama RA Kingston P Mustafa N Johnson E Pannell R Impact of an integrated rapid response psychiatric liaison team on quality improvement and cost savings: the Birmingham RAID model. Psychiatrist 2013; 37: 4–10.

[3] Desan PH Zimbrean PC Weinstein AJ Bozzo JE Sledge WH Proactive psychiatric consultation services reduce length of stay for admissions to an inpatient medical team. Psychosomatics 2011; 52: 513–20. 22054620 10.1016/j.psym.2011.06.002

[4] Lyons JS Hammer JS Strain JJ Fulop G The timing of psychiatric consultation in the general hospital and length of hospital stay. Gen Hosp Psychiatry 1986; 8: 159–62. 3710148 10.1016/0163-8343(86)90074-5

[5] Kishi Y Meller W Kathol R Swigart S Factors affecting the relationship between the timing of psychiatric consultation and general hospital length of stay. Psychosomatics 2004; 45: 470–6. 15546823 10.1176/appi.psy.45.6.470

[6] Alhuthail Y Timing of referral to consultation-liaison psychiatry. Int J Health Sci 2009; 3: 165–70. PMC3068806 21475534

[7] Bourgeois JA Wegelin JA Lagtime in psychosomatic medicine consultations for cognitive-disorder patients: association with length of stay. Psychosomatics 2009; 50: 622–5. 19996234 10.1176/appi.psy.50.6.622

[8] Ormont MA Weisman HW Heller SS Najara JE Shindledecker RD The timing of psychiatric consultation requests: utilization, liaison, and diagnostic considerations. Psychosomatics 1997; 38: 38–44. 8997115 10.1016/S0033-3182(97)71502-0

[9] Strain JJ Lyons JS Hammer JS Fahs M Lebovits A Paddison PL Cost offset from a psychiatric consultation-liaison intervention with elderly hip fracture patients. Am J Psychiatry 1991; 148: 1044–9. 1853954 10.1176/ajp.148.8.1044

[10] Handrinos D McKenzie D Smith GC Timing of referral to a consultation liaison psychiatry unit. Psychosomatics 1998; 39: 311–7. 9691700 10.1016/S0033-3182(98)71319-2

[11] Ackerman AD Lyons JS Hammer JS Larson DB The impact of coexisting depression and timing of psychiatric consultation on medical patients’ length of stay. Hosp Community Psychiatry 1988; 39: 173–6. 3345980 10.1176/ps.39.2.173

[12] Jonge PD Huyse FJ Ruinemans GM-F Stiefel FC Lyons JS Slaets JP Timing of psychiatric consultations: the impact of social vulnerability and level of psychiatric dysfunction. Psychosomatics 2000; 41: 505–11. 11110114 10.1176/appi.psy.41.6.505

[13] Royal College of Psychiatrists. Who Cares Wins. Improving the Outcome for Older People Admitted to the General Hospital. Guidelines for the Development of Liaison Mental Health Services for Older People. Royal College of Psychiatrists, 2005.

[14] Diefenbecher A Strain JJ Consultation-liaison psychiatry: stability and change over a 10-year-period. Gen Hosp Psychiatry 2002; 24: 249–56. 12100835 10.1016/s0163-8343(02)00182-2

[15] Rajmohan V Kumar SK Psychiatric morbidity, pain perception, and functional status of chronic pain patients in palliative care. Indian J Pall Care 2013; 19: 146–51. 10.4103/0973-1075.121527 PMC3853392 24347904

[16] American Psychiatric Association. Diagnostic and Statistical Manual of Mental Disorders (4th edn, revised) (DSM-IV-TR). APA, 2000.

[17] Saravay SM Steinberg MD Weinschel B Pollack S Alovis N Psychological comorbidity and length of stay in the general hospital. Am J Psychiatry 1991; 148: 324–9. 1992834 10.1176/ajp.148.3.324

[18] Hales RE Polly S Orman D An evaluation of patients who received an organic mental disorder diagnosis on a psychiatric consultation-liaison service. Gen Hosp Psychiatry 1988; 11: 88–94. 2707592 10.1016/0163-8343(89)90051-0

[19] Sydney Local Health District. Canterbury Hospital Strategic Plan 2013-2018. NSW Health, 2013 (http://www.slhd.nsw.gov.au/pdfs/Cant_StrategicPlan.pdf).

[20] Abernethy AP Shelby-James T Fazekas BS Woods D Currow DC The Australia-modified Karnofsky Performance Status (AKPS) scale: a revised scale for contemporary palliative care clinical practice. BMC Palliat Care 2005; 4: 7. 16283937 10.1186/1472-684X-4-7 PMC1308820

[21] Wand AP Thoo W Ting V Baker J Sciuriaga H Hunt GE Identification and rates of delirium in elderly medical inpatients from diverse language groups. Geri Nurs 2013; 34: 355–60. 10.1016/j.gerinurse.2013.05.004 23769870

[22] Wand AP Thoo W Sciuriaga H Ting V Baker J Hunt GE A multifaceted educational intervention to prevent delirium in older inpatients: a before and after study. Int J Nurs Stud 2014; 51: 947–82. 10.1016/j.ijnurstu.2013.11.005 24332570

[23] Kaboli PJ Go JT Hockenberry J Glasgow JM Johnson SR Rosenthal GE Associations between reduced hospital length of stay and 30-Day readmission rate and mortality: 14-year experience in 129 veterans affairs hospitals. Ann Intern Med 2012; 157: 837–45. 23247937 10.7326/0003-4819-157-12-201212180-00003

[24] Pincus HA Psychiatric consultations and length of hospital stay. Psychosomatics 2005; 46: 496. 16145196 10.1176/appi.psy.46.5.496

